# Severity, duration, and pattern of intraoperative hypotension and early postoperative cognitive decline in adults undergoing non-cardiac surgery: a prospective observational cohort study

**DOI:** 10.3389/fmed.2026.1972338

**Published:** 2026-10-28

**Authors:** Joud Firas Alnajjar, Ghazi Gasmalla, Diaaeldein Mahmoud, Marwan Ismail, Ellen Safadi

**Affiliations:** 1 Department of Anesthesia Technology, College of Health Sciences, Gulf Medical University, Ajman, United Arab Emirates; 2 Department of Anaesthesiology, Thumbay University Hospital, Ajman, United Arab Emirates; 3 Department of Medical Laboratory Sciences, College of Health Sciences, Gulf Medical University, Ajman, United Arab Emirates

**Keywords:** general anaesthesia, intraoperative hypotension, mean arterial pressure, mini-mental state examination, Montreal cognitive assessment, perioperative neurocognitive disorders, postoperative cognitive dysfunction

## Abstract

**Introduction:**

Intraoperative hypotension (IOH) is known to pose risks for myocardial and renal injury, but its links to early postoperative cognitive decline (ePOCD) are ambiguous due to varying definitions of IOH across studies.

**Method:**

This prospective observational cohort study at a tertiary teaching hospital in Ajman, United Arab Emirates, involved 261 adults undergoing elective non-cardiac surgeries under general anaesthesia. The study defined IOH as a decrease in mean arterial pressure (MAP) exceeding 20% from the pre-induction baseline and categorised it by severity (mild, moderate, severe) and duration/pattern (controlled episodes, prolonged episodes, frequent short episodes). Cognitive assessment was conducted using the Mini-Mental State Examination and Montreal Cognitive Assessment pre-surgery, immediately after, and on postoperative day 1.

**Results:**

The results indicated that 93.1% of patients experienced IOH as defined, primarily mild and moderate cases, with no severe instances recorded. ePOCD was identified in 3.1% of patients. Scores on both cognitive assessments dropped significantly right after surgery but showed partial recovery by day 1. Notably, the presence of IOH did not correlate with ePOCD, whereas the severity and pattern of hypotension were associated with it, with higher ePOCD rates in patients with moderate hypotension and in those with a single prolonged episode or frequent short episodes than in those with a single short (controlled) episode. In addition, patients experiencing ePOCD were significantly older and had longer durations of hypotension. The findings suggest that a simple threshold of >20% relative change in MAP fails to predict ePOCD risk, whereas cumulative exposure, severity, and pattern of hypotension were associated with ePOCD in this exploratory analysis. Because all ePOCD events occurred in the oldest patients, these associations could not be separated from the effect of age and should not be interpreted as evidence that these measures predict ePOCD independently.

**Discussion:**

These results argue against relying on binary definitions of IOH in neurocognitive studies and highlight the importance of exposure metrics. Confounding, particularly by age, prevented causal inference because of the small number of ePOCD events and collinearity between age and hypotensive exposure.

## Introduction

Perioperative neurocognitive disorders are among the most consequential complications of surgery and anaesthesia, and they are associated with prolonged recovery, loss of functional independence, and increased long-term mortality (1–3). Under the 2018 consensus nomenclature, cognitive change identified within 30 days of surgery is classified as delayed neurocognitive recovery, whereas the term postoperative cognitive dysfunction (POCD), or postoperative neurocognitive disorder, is reserved for impairment persisting between 30 days and 12 months (1, 2). This distinction matters, because the determinants of transient early decline and of persistent impairment are unlikely to be identical. In line with this framework, the present study reports early postoperative cognitive decline (ePOCD) and does not claim to measure POCD as defined by the consensus statement.

Intraoperative hypotension is the most common haemodynamic disturbance of general anaesthesia and, unlike most risk factors for neurocognitive injury, it is directly modifiable (4, 5). Large observational cohorts have established a graded relationship between the depth and duration of intraoperative hypotension and myocardial injury, acute kidney injury, and mortality after non-cardiac surgery (6–9). The cerebral analogue of this relationship is biologically plausible: when MAP falls below the lower limit of cerebral autoregulation, cerebral blood flow becomes pressure-passive, and ageing and chronic hypertension shift that limit rightwards, narrowing the margin of safety (10, 11).

The clinical evidence, however, is inconsistent. A randomised pilot trial of differing intraoperative blood pressure strategies found no association with postoperative cognitive dysfunction (12), and a meta-analysis of randomised trials reached a similar conclusion while noting substantial heterogeneity and limited power (13). Conversely, observational work has linked hypotensive burden to postoperative neurocognitive disorders and to delirium (14–17).

A major and under-acknowledged source of this inconsistency is definitional. More than 140 distinct definitions of intraoperative hypotension have been used in the adult non-cardiac surgical literature, and the reported incidence in the same patients varies from approximately 5% to 99% depending on which is chosen (4, 5). Definitions based on a relative fall from baseline, absolute thresholds, and time- or area-weighted exposure metrics are not interchangeable, and the consequences of this choice for neurocognitive endpoints have rarely been examined directly.

Data from the Gulf region is also sparse. Surgical populations in the United Arab Emirates are comparatively young and ethnically diverse, and it is not known whether relationships derived from older European and North American cohorts transfer to this setting.

Therefore, we conducted a prospective observational cohort study with three aims: to quantify the incidence, severity, duration, and pattern of intraoperative hypotension in adults undergoing non-cardiac surgery under general anaesthesia; to determine the incidence of early postoperative cognitive decline measured with the Mini-Mental State Examination (MMSE) and Montreal Cognitive Assessment (MoCA); and to examine whether the occurrence of hypotension, as opposed to its severity, duration, and pattern, is associated with that outcome.

## Methods

### Study design

This was a single-centre prospective observational cohort study.

### Setting and participants

The study was conducted at Thumbay University Hospital, a tertiary care teaching hospital of Gulf Medical University in Ajman, United Arab Emirates, serving both Emirati nationals and a large expatriate population. Recruitment took place between March and June 2026.

Consecutive adults scheduled for elective non-cardiac surgery under general anaesthesia were screened for eligibility. The inclusion criteria were age 18 years or older, general anaesthesia for a non-cardiac procedure, capacity to give informed consent, and ability to communicate in English or Arabic. The exclusion criteria were pre-existing cognitive impairment or dementia, neurological disease affecting cognition, neurosurgical procedures, refusal to participate, and postoperative complications precluding cognitive reassessment. Patients discharged on the day of surgery were withdrawn because the day 1 assessment could not be obtained. Pre-existing cognitive impairment was screened for before enrolment by review of the electronic medical record and a structured preoperative interview with the patient (and an accompanying family member where available) for any documented diagnosis of dementia, mild cognitive impairment or other cognitive disorder, or current use of anti-dementia medication; patients with a preoperative MMSE score below 24 or MoCA score below 26 were also excluded.

### Sample size

The accessible population was estimated from an institutional throughput of approximately 2,300 operations annually, giving roughly 575 eligible patients over a 3-month recruitment window. Using a single-population proportion formula with *p* = 0.5, a 95% confidence level, and a 5% margin of error, an unadjusted sample of 384 was required; applying a finite population correction for *N* = 575 yielded 230, and adding 10% for incomplete data produced a target of approximately 250 participants. This calculation was designed to estimate the prevalence of exposure and outcome with acceptable precision and was not powered to detect an association between hypotensive exposure and cognitive decline; the implications are addressed in the Limitations section.

### Exposure definition and measurement

Blood pressure was monitored throughout the surgical procedure in accordance with the American Society of Anesthesiologists (ASA) standards for basic anaesthetic monitoring. Blood pressure was measured non-invasively using an automated oscillometric blood pressure monitor at 5-minute intervals. No patient underwent invasive arterial blood pressure monitoring. Baseline mean arterial pressure (MAP) was defined as the MAP value recorded before induction of anaesthesia. Intraoperative hypotension was defined *a priori* as any recorded MAP value more than 20% below the individual patient's pre-induction baseline MAP. Three additional exposure characterisations were recorded:

- *Severity*, from the maximal proportional MAP reduction: no IOH (≤20%), mild (20.1%–30%), moderate (30.1%–40%), and severe (>40%).
- *Cumulative duration*—the total number of minutes for which MAP was below the individual threshold. Because MAP was measured every 5 min, hypotensive time was derived from the intermittent readings. Consecutive readings below the individual threshold were treated as one hypotensive episode, and episodes separated by at least one reading at or above the threshold were counted as separate episodes. The start and end of each episode were estimated by linear interpolation between the last reading above and the first reading below the threshold (and vice versa), assuming a linear change in MAP between readings; episode duration was the time between these estimated crossing points, and cumulative duration was the sum of all episode durations. Durations are therefore not restricted to multiples of 5 min. The ≤10 versus >10 min classification used to define hypotension pattern was based on these estimated episode durations. Patients without hypotension were assigned a cumulative duration of 0 min.
- *Hypotension Pattern*. The pattern of intraoperative hypotension was classified *a priori* according to the duration and frequency of hypotensive episodes. A single short (controlled) episode was defined as one hypotensive episode lasting ≤10 min. A single prolonged episode was defined as one continuous hypotensive episode lasting >10 min. Patients experiencing two or more separate hypotensive episodes, each lasting ≤10 min, were classified as having frequent short episodes. The lowest recorded intraoperative MAP was also documented for each patient.
- *Intraoperative Management Variables.* Information regarding intraoperative vasopressor administration, intravenous fluid therapy, anaesthetic agents, and depth-of-anaesthesia monitoring were available in the anaesthesia records but were not extracted or analysed as study variables. The study was specifically designed to investigate the association between the severity, duration, and pattern of intraoperative hypotension and early postoperative cognitive decline. Intraoperative haemodynamic and anaesthetic management was determined by the attending anaesthesiologist according to the clinical requirements of each patient. Intravenous fluids were administered as clinically indicated, with Ringer's lactate routinely used. Therefore, specific vasopressor types, doses, and timing; anaesthetic agents and their doses; intravenous fluid volumes and timing; and depth-of-anaesthesia-monitoring parameters were not included in the present analysis.
- *Outcome Definition and Measurement*. Cognitive function was assessed using the MMSE and MoCA, both 30-point instruments with established psychometric properties (18, 19). Assessments were performed preoperatively, immediately after surgery once the patient was awake and able to communicate, and on postoperative day 1. Early postoperative cognitive decline was defined *a priori* as a > 2-point decrease from the individual preoperative baseline on either the MMSE or the MoCA. This threshold was chosen because published test–retest, reliable-change and minimum-detectable-change data for both instruments indicate that changes of 2 points or less cannot be reliably distinguished from measurement error and practice effects in an individual patient (20, 21). A decline of more than 2 points was therefore taken as the smallest change likely to represent a true within-person decline. Because baseline scores in this cohort were close to the instrument ceiling, this threshold is conservative, and we acknowledge that other cut-offs, or a reliable change index derived from a non-surgical control group, could classify some patients differently. Primary outcome classification was based on the immediate postoperative assessment, while the postoperative day 1 assessment was used to evaluate subsequent cognitive performance. The same test versions were used at all time points. All assessments were performed by the same researcher, who was fluent in English and Arabic and familiar with both instruments; administration procedures were reviewed with a clinical psychologist before the study. The assessor was not blinded to intraoperative haemodynamic data. When clarification or translation was required, an electronic translation tool was used.
- *Covariates*. Age, sex, documented medical and surgical history, procedure type, and duration of surgery were recorded using a structured case report form. All participants were classified as ASA physical status I or II, and none had an educational level below middle school. Patients receiving medications considered likely to affect postoperative cognitive function were excluded. These criteria were applied to reduce potential confounding factors that could influence postoperative cognitive assessment.
- *Statistical analysis*. Analyses were performed in IBM SPSS Statistics version 30 (IBM Corp., Armonk, NY, USA). Categorical variables were presented as frequencies and percentages with 95% Wilson confidence intervals for key proportions; continuous variables as mean ± standard deviation and median with range. Normality was assessed by the Shapiro–Wilk test; because all continuous variables departed significantly from normality (Additional file 2), non-parametric methods were used throughout.

Within-person cognitive change was tested using the Wilcoxon signed-rank test and between-group differences with the Mann–Whitney *U* test; for both, the effect size *r* was calculated as |*Z*|/√*N* and interpreted using conventional thresholds (0.10 small, 0.30 medium, 0.50 large). Associations between categorical exposures and the outcome were tested using Fisher's exact test for 2 × 2 tables and the Freeman–Halton extension for larger tables, because expected cell counts were below 5 in every contingency table; the corresponding Pearson chi-square values are reported for completeness but are not used for inference. An ordered dose–response across severity strata was tested using the Cochran–Armitage test for trend. Correlations between continuous exposure metrics and cognitive change scores used Spearman's rho.

Binary logistic regression was performed but, with only eight outcome events, the number of events per variable was far below the accepted minimum of 10, and the models were reported as exploratory and hypothesis-generating only. Age could not be entered because outcome events were confined to the oldest patients, producing quasi-complete separation and unstable maximum likelihood estimates. Two-sided *p*-values below 0.05 were considered statistically significant; because multiple exposure characterisations were examined, *p*-values were interpreted descriptively and no formal correction for multiplicity was applied.

### Ethical approval

This study was approved by the Institutional Review Board of Gulf Medical University on 12 February 2026 (reference IRB-COHS-STD-98-Feb-2026) and was conducted in accordance with the Declaration of Helsinki. Written informed consent was obtained from all participants, who were informed that participation was voluntary and that refusal or withdrawal would not affect their care. Identifiers were replaced by study codes and electronic data were held in password-protected files accessible only to the research team.

## Results

### Participants

Adult patients scheduled for elective non-cardiac surgery under general anaesthesia were assessed for eligibility. Patients were excluded if they had a language barrier that prevented reliable communication or standardised administration of the MMSE and MoCA; declined participation; were unable to participate because of anxiety or fear; were discharged before completion of the postoperative day 1 assessment; declined or were unable to cooperate with postoperative cognitive assessment; or underwent a change in the planned anaesthetic technique from general to spinal anaesthesia. Patients receiving medications considered likely to affect postoperative cognitive function, or meeting other prespecified protocol exclusion criteria, were also excluded.

Approximately 60 patients were excluded during the screening process for the above reasons. A total of 261 eligible participants who completed the required cognitive assessments were included in the final analysis. The screening, exclusions, and final study population are shown in the participant flow diagram (Figure 1).

**Figure 1:**
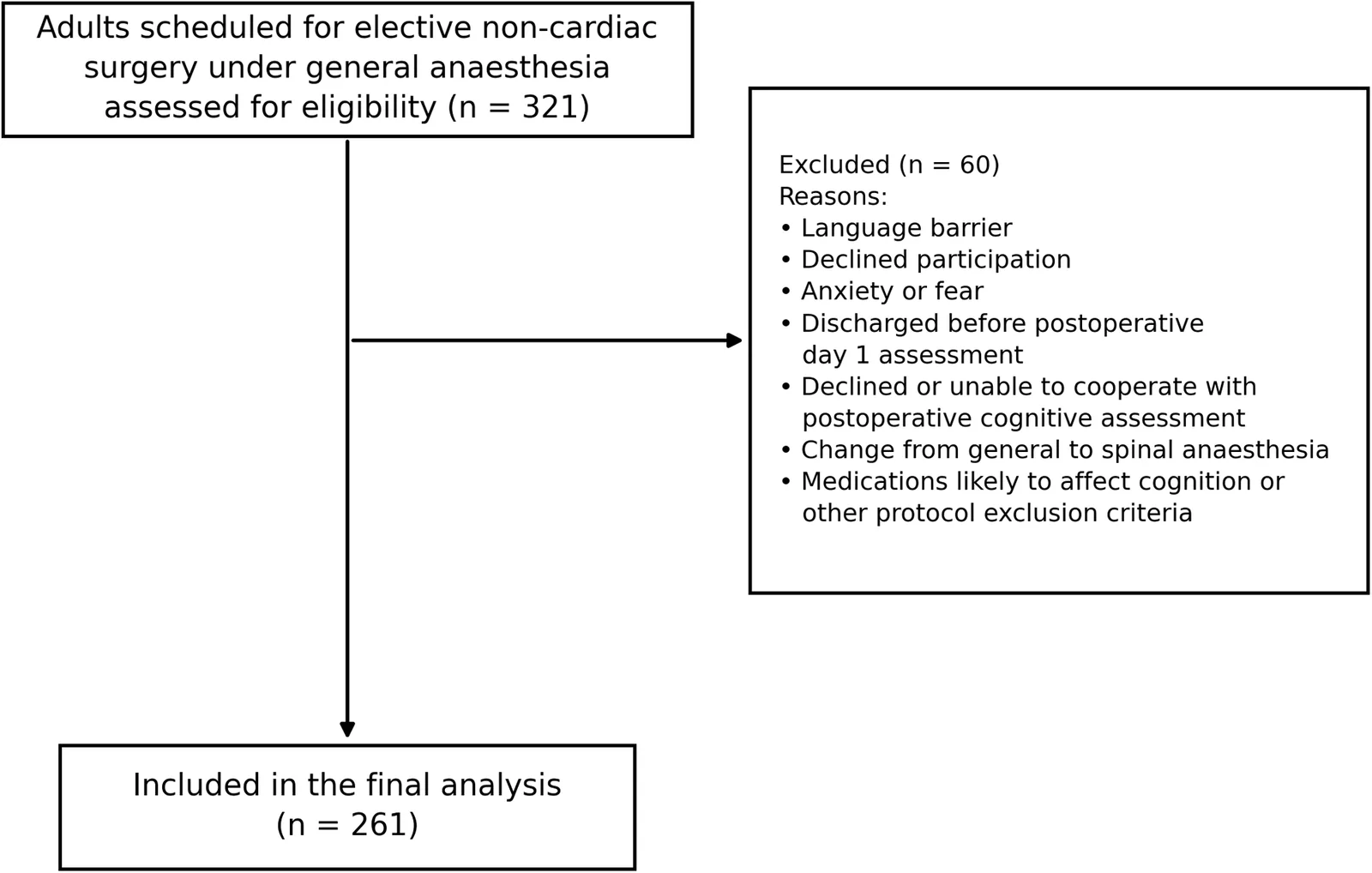
A participant flow diagram. Number of patients assessed for eligibility, excluded before enrolment (with reasons) and included in the final analysis (*n* = 261).

The 261 analysed patients had a mean age of 42.6 ± 13.6 years (median 41, range 21–79); 134 (51.3%) were men. Only 37 patients (14.2%) were aged 60 years or older and 5 (1.9%) were aged 70 or older. No previous medical or surgical history was documented in 105 patients (40.2%); hypertension (12.6%) and type 2 diabetes (9.2%) were the most frequently recorded conditions. Procedures were predominantly short and of low to moderate physiological severity, with a mean surgical duration of 87.2 ± 30.3 min (Table 1).

**Table 1:** Baseline characteristics of the cohort (*n* = 261).

Characteristic	*n* (%) or mean ± SD
Age (years)	42.6 ± 13.6; median 41 (range 21–79)
18–29	56 (21.5)
30–39	61 (23.4)
40–49	60 (23.0)
50–59	47 (18.0)
60–69	32 (12.3)
≥70	5 (1.9)
Sex	
Male	134 (51.3)
Female	127 (48.7)
Documented previous medical or surgical history	
None	105 (40.2)
Hypertension	33 (12.6)
Previous appendectomy	25 (9.6)
Type 2 diabetes mellitus	24 (9.2)
Previous caesarean section	20 (7.7)
Asthma	19 (7.3)
Previous ENT surgery	18 (6.9)
Previous laparoscopy	17 (6.5)
Surgical specialty	
General and gastrointestinal	99 (37.9)
Ear, nose, and throat	73 (28.0)
Urological	55 (21.1)
Gynaecological	27 (10.3)
Orthopaedic and spinal	4 (1.5)
Ophthalmic	3 (1.1)
Duration of surgery (min)	87.2 ± 30.3; median 83 (range 29–249)

ENT, ear, nose, and throat. Previous history was recorded as a single category per patient; total comorbidity burden was not captured. All patients were ASA physical status I or II*.*

### Intraoperative haemodynamics

Baseline MAP was 95.2 ± 4.4 mmHg, and the lowest recorded intraoperative MAP was 68.7 ± 6.3 mmHg, corresponding to a mean maximal reduction of 27.9 ± 5.6% (Table 2). Applying the prespecified relative threshold, 243 of 261 patients (93.1%, 95% CI 89.4–95.6) were classified as having intraoperative hypotension. Reductions were mild in 168 patients (64.4%) and moderate in 75 (28.7%); no patient exceeded the 40% threshold for severe hypotension. Among the 243 patients with hypotension, cumulative hypotension duration was short in most (mean 9.5 ± 6.0 min; median 8, range 4–40). Of these 243 patients, 221 (90.9%) had a single controlled episode, 20 (8.2%) had a single prolonged episode, and 2 (0.8%) had frequent short episodes; the 18 patients without hypotension were classified separately.

**Table 2:** Intraoperative haemodynamic exposure (*n* = 261).

Exposure variable	Value
Baseline mean arterial pressure (mmHg)	95.2 ± 4.4; median 95 (range 88–108)
Lowest intraoperative mean arterial pressure (mmHg)	68.7 ± 6.3; median 69 (range 54–82)
Maximal reduction in mean arterial pressure (%)	27.9 ± 5.6; median 27 (range 20–39)
Cumulative duration of hypotension, patients with IOH (*n* = 243) (min)	9.5 ± 6.0; median 8 (range 4–40)
Intraoperative hypotension (>20% reduction)	
Absent	18 (6.9)
Present	243 (93.1); 95% CI 89.4–95.6
Severity of reduction	
No hypotension (≤20%)	18 (6.9)
Mild (20.1%–30%)	168 (64.4)
Moderate (30.1%–40%)	75 (28.7)
Severe (>40%)	0 (0.0)
Pattern of hypotension	
No hypotension	18 (6.9)
Controlled single episode	221 (84.7)
Single prolonged episode	20 (7.7)
Frequent short episodes	2 (0.8)

Values are mean ± SD with median (range), or *n* (%).

### Cognitive trajectory

Preoperative scores were high and close to the instrument ceiling (MMSE 29.51 ± 0.54; MoCA 28.95 ± 0.87). Both instruments declined significantly immediately after surgery and remained significantly below baseline on day 1, with all four comparisons reaching *p* < 0.001 and large rank-based effect sizes (*r* = 0.56–0.74). The absolute magnitude of change was nevertheless small, with mean declines of 0.64 and 0.57 points immediately after surgery for the MMSE and MoCA, respectively, falling to 0.37 and 0.34 points by day 1 (Table 3, Figure 2). Change was unidirectional: across all four comparisons, there was not a single patient whose postoperative score exceeded baseline, and between 43% and 67% of patients showed no change at all.

**Table 3:** Cognitive scores and within-person change (*n* = 261).

Comparison	Preoperative	Postoperative	Mean decline	*Z*	*p*	*r*
MMSE: preoperative vs. immediate postoperative	29.51 ± 0.54	28.87 ± 0.94	0.64	−11.911	<0.001	0.74
MMSE: preoperative vs. day 1	29.51 ± 0.54	29.14 ± 0.84	0.37	−9.359	<0.001	0.58
MoCA: preoperative vs. immediate postoperative	28.95 ± 0.87	28.37 ± 1.24	0.57	−11.090	<0.001	0.69
MoCA: preoperative vs. day 1	28.95 ± 0.87	28.60 ± 1.08	0.34	−8.980	<0.001	0.56

Wilcoxon signed-rank test. *r* = |*Z*|/√*N*. No patient scored above baseline at any postoperative time point; the number of tied (unchanged) observations was 112, 169, 131, and 176, respectively. MMSE, Mini-Mental State Examination; MoCA, Montreal Cognitive Assessment.

**Figure 2:**
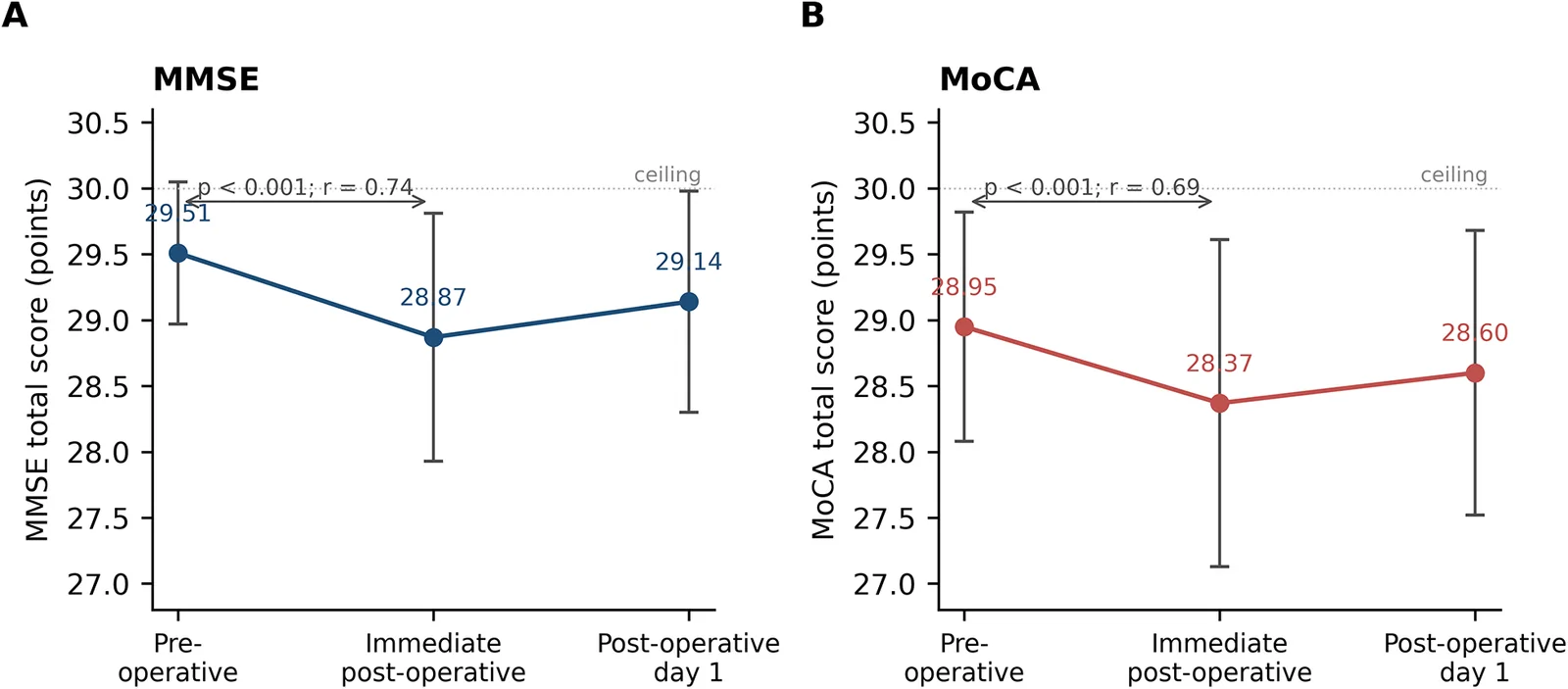
Cognitive scores before and after surgery (*n* = 261). **(A)** Mini-Mental State Examination (MMSE) and **(B)** Montreal Cognitive Assessment (MoCA) total scores before surgery, immediately after surgery, and on postoperative day 1. The small grey dots show individual patient scores, jittered horizontally because scores are whole numbers; large coloured points show the means, and the error bars show ±1 standard deviation (SD). The dotted line marks the instrument ceiling of 30 points. The *p*-value and effect size (*r*) shown are for the Wilcoxon signed-rank comparison of preoperative with immediate postoperative scores.

### Early postoperative cognitive decline

Eight of 261 patients met the outcome definition, an incidence rate of 3.1% (95% CI 1.6–5.9; exact 95% CI 1.3–6.0).

### Association between hypotensive exposure and cognitive decline

The dichotomous presence of intraoperative hypotension was not associated with the outcome. All 8 events occurred among the 243 exposed patients (3.3%) and none among the 18 unexposed patients (0%), but this difference was well within chance (Fisher's exact *p* = 1.000; Table 4).

**Table 4:** Association between intraoperative hypotension and early postoperative cognitive decline.

Exposure	No decline	Decline	Rate (%)	Exact p
Occurrence				1.000^a^
No hypotension (≤20% reduction)	18	0	0.0	
Hypotension (>20% reduction)	235	8	3.3	
Severity				0.002^b^
No hypotension (≤20%)	18	0	0.0	
Mild (20.1%–30%)	167	1	0.6	
Moderate (30.1%–40%)	68	7	9.3	
Pattern				<0.001^b^
No hypotension (≤20% reduction)	18	0	0.0	
Controlled single episode	221	0	0.0	
Single prolonged episode	14	6	30.0	
Frequent short episodes	0	2	100.0	

a Fisher's exact test.

b Freeman–Halton exact test. Cochran–Armitage test for trend across severity strata: *z* = 3.40, *p* = 0.001. Expected counts were below 5 in every table, and hence, chi-square values are not used for inference. Unadjusted odds ratio for moderate versus mild severity 17.0 (95% CI 2.1–779).

Severity showed a clear monotonic gradient. The event rate was 0% (0/18) with no hypotension, 0.6% (1/168, 95% CI 0.1–3.3) with mild hypotension, and 9.3% (7/75, 95% CI 4.6–18.0) with moderate hypotension (Freeman–Halton exact *p* = 0.002; Cochran–Armitage trend *p* = 0.001; Figure 3A). The unadjusted odds ratio for moderate versus mild hypotension was 17.0, although the confidence interval was extremely wide (95% CI 2.1–779), reflecting the sparse cell counts.

**Figure 3:**
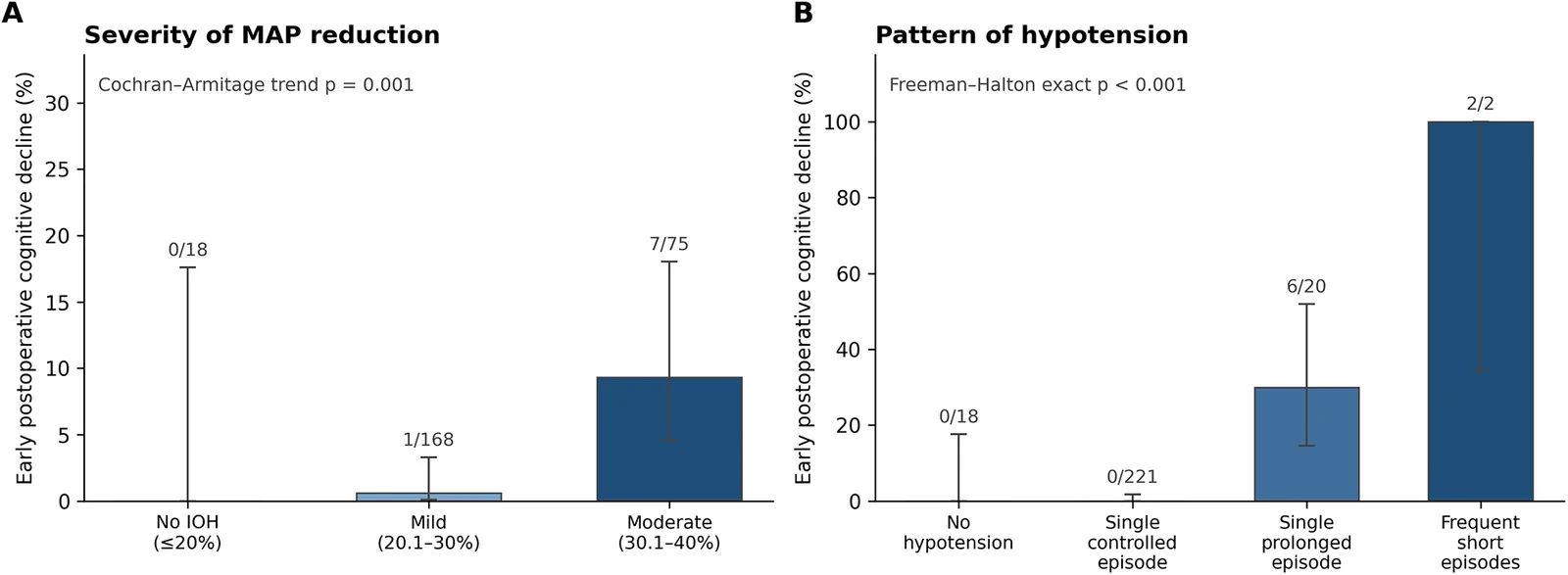
Incidence of early postoperative cognitive decline by severity and pattern of intraoperative hypotension. **(A)** By severity of the maximal proportional reduction in mean arterial pressure: no hypotension (≤20%), mild (20.1%–30%), and moderate (30.1%–40%); no patient had severe (>40%) hypotension. **(B)** By temporal pattern: no hypotension (*n* = 18), single controlled episode of ≤10 min (*n* = 221), single prolonged episode of >10 min (*n* = 20), and frequent short episodes (two or more episodes, each ≤10 min; *n* = 2). The bars show the observed percentage of patients with early postoperative cognitive decline, and the error bars show 95% Wilson confidence intervals. The numbers above each bar are events/patients in that group. The *p*-values are derived from the Cochran–Armitage test for trend **(A)** and the Freeman–Halton exact test **(B)**

Pattern showed the strongest association. No patient with a controlled single hypotensive episode developed cognitive decline (0/221), and none of the 18 patients without hypotension did so (0/18). By contrast, cognitive decline occurred in six of 20 patients (30.0%) with a single prolonged episode and both patients with frequent short episodes (Freeman–Halton exact *p* < 0.001; Figure 3B). Because pattern and cumulative duration are partly different expressions of the same underlying exposure, these two analyses should not be regarded as independent findings.

### Comparison of patients with and without cognitive decline

Patients who developed early cognitive decline differed from those who did not on several dimensions (Table 5). The largest difference was in age: 71.5 ± 4.4 years versus 41.7 ± 12.7 years (*p* < 0.001, *r* = 0.30). All 8 events occurred in the oldest segment of the cohort. Cumulative hypotension duration was also markedly longer in patients with cognitive decline (33.0 ± 7.9 versus 8.8 ± 4.2 min, *p* < 0.001, *r* = 0.30), as was the proportional MAP reduction (33.5 ± 3.4% versus 27.7 ± 5.5%, *p* = 0.003) and baseline MAP (103.3 ± 4.2 versus 95.0 ± 4.2 mmHg, *p* < 0.001). The lowest intraoperative MAP did not differ between the groups (68.6 ± 5.0 versus 68.7 ± 6.3 mmHg, *p* = 0.792), and surgical duration differed only marginally (*p* = 0.056).

**Table 5:** Comparison of patients with and without early postoperative cognitive decline.

Variable	Decline (*n* = 8)	No decline (*n* = 253)	*Z*	*p*	*r*
Age (years)	71.5 ± 4.4	41.7 ± 12.7	4.813	<0.001	0.30
Duration of surgery (min)	125.4 ± 68.8	86.0 ± 27.7	1.908	0.056	0.12
Baseline mean arterial pressure (mmHg)	103.3 ± 4.2	95.0 ± 4.2	3.900	<0.001	0.24
Lowest mean arterial pressure (mmHg)	68.6 ± 5.0	68.7 ± 6.3	−0.264	0.792	0.02
Maximal reduction in mean arterial pressure (%)	33.5 ± 3.4	27.7 ± 5.5	3.012	0.003	0.19
Cumulative duration of hypotension (min)	33.0 ± 7.9	8.8 ± 4.2	4.762	<0.001	0.30
MMSE decline, immediate postoperative (points)	3.12 ± 0.64	0.56 ± 0.50	5.507	<0.001	0.34
MMSE decline, day 1 (points)	1.62 ± 0.52	0.33 ± 0.47	5.066	<0.001	0.31
MoCA decline, immediate postoperative (points)	3.50 ± 0.76	0.48 ± 0.50	5.478	<0.001	0.34
MoCA decline, day 1 (points)	1.62 ± 0.52	0.30 ± 0.46	5.228	<0.001	0.32

Mann–Whitney *U* test; *r* = |*Z*|/√*N*. Cognitive decline variables are components of the outcome definition and are shown for description only and not as independent predictors.

**Table 6:** Logistic regression for early postoperative cognitive decline (exploratory).

Predictor	*B*	SE	Wald	*p*	OR	95% CI
Unadjusted models						
Cumulative duration of hypotension (per min)	0.372	0.106	12.240	<0.001	1.45	1.18–1.79
Baseline mean arterial pressure (per mmHg)	0.688	0.203	11.477	<0.001	1.99	1.34–2.96
Maximal reduction in mean arterial pressure (per 1%)	0.191	0.072	7.068	0.008	1.21	1.05–1.39
Duration of surgery (per min)	0.028	0.009	9.488	0.002	1.03	1.01–1.05
Lowest mean arterial pressure (per mmHg)	−0.002	0.057	0.001	0.978	1.00	0.89–1.12
Two-variable model						
Cumulative duration of hypotension (per min)	0.371	0.117	10.095	0.001	1.45	1.15–1.82
Maximal reduction in mean arterial pressure (per 1%)	0.108	0.116	0.876	0.349	1.12	0.89–1.40

With eight outcome events, the events-per-variable ratio was 8 (one predictor) and 4 (two predictors), below the accepted minimum of 10. Age could not be entered because of quasi-complete separation. A two-variable model—Nagelkerke R^2^ 0.81 and Hosmer–Lemeshow *p* 1.000—is a pattern indicating near separation rather than good calibration. All estimates are only exploratory. OR odds ratio; CI, confidence interval.

### Correlation and exploratory regression

Across the whole cohort, correlations between continuous hypotension metrics and cognitive change scores were negligible, with only cumulative duration and day 1 MoCA change reaching nominal significance (rho=0.126, *p* = 0.042; Additional file 3). This apparent discrepancy with the group comparison is expected: the association is driven by a small number of extreme observations rather than by a graded relationship across the bulk of the cohort, in which cognitive change was near zero.

In unadjusted logistic regression, cumulative hypotension duration (OR 1.45 per minute, 95% CI 1.18–1.79), baseline MAP (OR 1.99 per mmHg, 95% CI 1.34–2.96), proportional MAP reduction (OR 1.21 per 1%, 95% CI 1.05–1.39), and surgical duration (OR 1.03 per minute, 95% CI 1.01–1.05) were each associated with the outcome, whereas the lowest MAP was not (OR 1.00, 95% CI 0.89–1.12) (Figure 4, Table 6). In a two-variable model, cumulative duration remained associated with the outcome (OR 1.45, 95% CI 1.15–1.82), while proportional MAP reduction did not (OR 1.12, 95% CI 0.89–1.40).

**Figure 4:**
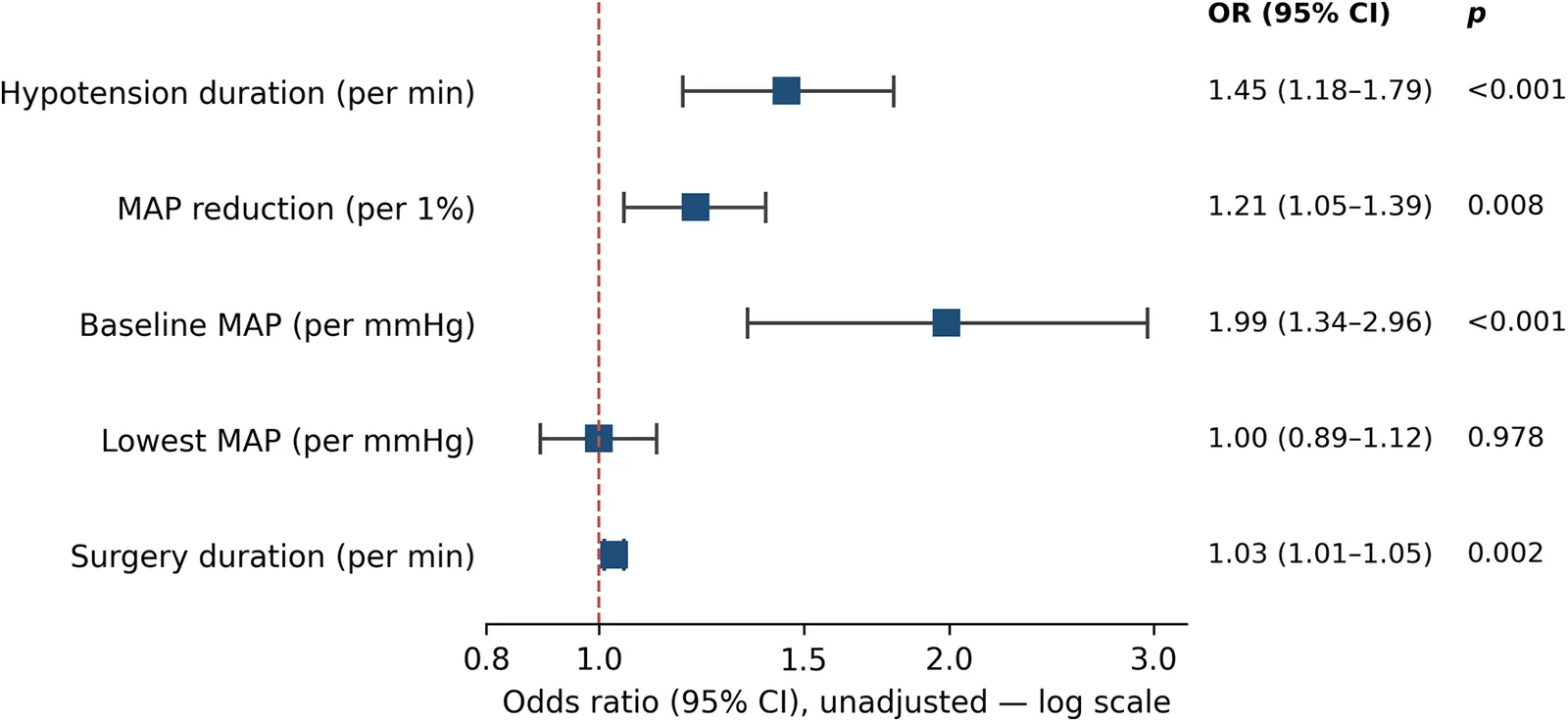
Unadjusted odds ratios for early postoperative cognitive decline from univariable logistic regression. The squares show the odds ratio for each predictor, and the horizontal error bars show the 95% confidence interval; the numerical values and *p*-values are listed on the right. The dashed vertical line marks an odds ratio of 1 (no association). The *x*-axis is on a logarithmic scale. Because only eight outcome events occurred, these estimates are only exploratory.

These models must be interpreted with great caution. With eight events, the events-per-variable ratio was 8 for a single predictor and 4 for two predictors, well below accepted thresholds; the Nagelkerke *R*^2^ of 0.81 and Hosmer–Lemeshow *p* of 1.000 in the two-variable model are characteristic of near-separation rather than of good fit, and age—the strongest single discriminator in the cohort—could not be included at all. The regression results are therefore reported as descriptive summaries of the exposure–outcome pattern and not as estimates of independent effect.

### Baseline dependence of the relative severity classification

Because the exposure was defined as a proportional fall from each patient's own pre-induction baseline, the severity classification is a function of both the nadir reached and the pressure from which it was measured. The two groups reached an almost identical nadir (68.6 ± 5.0 versus 68.7 ± 6.3 mmHg, *p* = 0.792) but differed substantially in pre-induction baseline MAP (103.3 ± 4.2 versus 95.0 ± 4.2 mmHg, *p* < 0.001), and therefore, the larger proportional reduction in patients with cognitive decline (33.5 ± 3.4% versus 27.7 ± 5.5%) is attributable to the baseline rather than to the depth of hypotension (Figure 5A). Recomputing the proportional reduction from the observed case-group nadir against the cohort mean baseline of 95.0 mmHg yields a rate of 27.7%, below the 30% cut-off, which would reclassify these patients from moderate to mild hypotension (Figure 5B). The same dependence applies to cumulative duration: the threshold below which hypotensive time accrued was itself 20% below each patient's baseline and was therefore 82.6 mmHg in patients with cognitive decline compared with 76.0 mmHg in the remainder.

**Figure 5:**
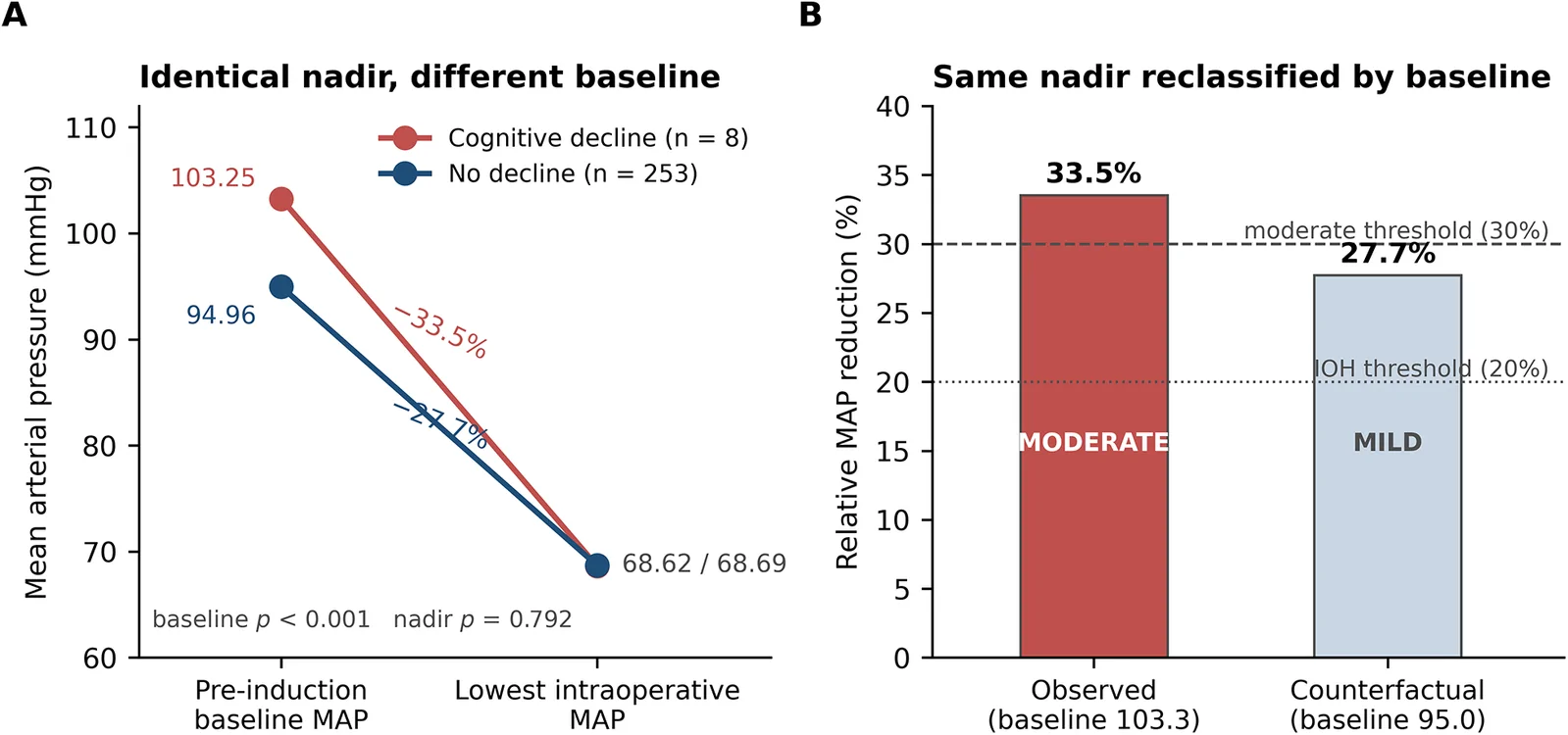
Dependence of the relative hypotension classification on pre-induction baseline pressure. **(A)** The pre-induction baseline and the lowest intraoperative mean arterial pressure (MAP) in patients with (*n* = 8, red) and without (*n* = 253, blue) early postoperative cognitive decline. The small dots show individual patients; the large points joined by the lines show the group means, and the percentages on the lines are the mean proportional reductions. The groups differ at baseline (*p* < 0.001) but not at the nadir (*p* = 0.792; Mann–Whitney *U* test). **(B)** Mean proportional MAP reduction observed in patients with cognitive decline (33.5%, from a mean baseline of 103.3 mmHg) compared with the value obtained for the same nadir measured from the cohort mean baseline of 95.0 mmHg (27.7%). The dashed line marks the 30% cut-off separating mild from moderate hypotension and the dotted line the 20% threshold defining intraoperative hypotension. Panel B is an arithmetic recomputation and not a statistical comparison; no error bars or *p*-values apply to it.

## Discussion

In this prospective cohort of 261 adults undergoing predominantly short, low-risk non-cardiac surgery, three findings stand out. First, a relative definition of intraoperative hypotension based on a >20% fall from baseline classified 93% of the cohort as exposed and had no discriminative value whatsoever for early cognitive decline. Second, when the same exposure was characterised by severity, cumulative duration, and temporal pattern, a consistent gradient emerged. Third, the patients in whom that gradient was expressed were almost exclusively the oldest patients in the cohort, and therefore, hypotensive exposure and age could not be disentangled.

### The definition of intraoperative hypotension is not a technicality

The first finding is, in our view, the most generalisable. A near-universal exposure cannot discriminate between patients who do and do not develop an outcome, whatever the underlying biology. Our observed 93% incidence rate sits at the upper end of the range documented in reviews of IOH definitions, in which incidence in identical patients varies from single figures to almost universal depending on the threshold applied (4, 5). Our data show empirically what those reviews predict: within a single cohort, the same haemodynamic record yields a null result under a dichotomous relative definition and a strongly graded result under duration- and pattern-based characterisation.

This has direct implications for the interpretation of existing literature. Studies reporting no association between intraoperative hypotension and neurocognitive outcomes (12, 13) have frequently used dichotomous definitions, and a null result under such a definition is weaker evidence of no effect than it appears. It also supports the movement in perioperative haemodynamic research towards dose-based exposure metrics—time below the threshold, area under the threshold, and time-weighted average MAP—which preserve the information that dichotomisation discards (6, 8, 9).

A second, less widely appreciated consequence of the relative definition is that it embeds the pre-induction baseline in the exposure variable itself. Patients who developed cognitive decline reached the same nadir as those who did not, and their apparently greater hypotensive severity was a function of their higher starting pressure (Figure 5). Because the threshold for accumulating hypotensive time is likewise set 20% below each patient's own baseline, the same absolute pressure trace generates a longer recorded duration in a patient with a high baseline than in one with a low baseline. Baseline MAP is therefore not an independent covariate in this design but a component of the exposure definition, and part of the severity and duration gradients we report is an arithmetic consequence of that construction. This does not dissolve the dose–response signal—the case group spent nearly four times as long below the threshold, which a 6.6 mmHg difference in the threshold is unlikely to explain in full—but a relative definition cannot separate the two. An absolute or area-under-threshold formulation, in which the same pressure means the same exposure in every patient, is a prerequisite for interpreting these data rather than merely a preferable convention.

A related observation is that the relative and absolute definitions were not interchangeable in our cohort. The mean lowest MAP was 68.7 mmHg, and the distribution implies that a substantial minority of patients fell below the conventional absolute threshold of 65 mmHg despite a comparatively modest proportional reduction, while others met the relative criterion without ever falling below 65 mmHg.

### Exposure dose, not exposure occurrence

Patients who developed early cognitive decline had spent a mean of 33 min below their individual hypotension threshold compared with under 9 min in those who did not, and no patient with a single controlled episode developed the outcome. This is congruent with the dose–dependent relationships established for myocardial and renal injury after non-cardiac surgery (6–9) and with observational reports linking hypotensive burden to postoperative delirium and neurocognitive disorders (14–17). Mechanistically, sustained rather than transient reduction in cerebral perfusion pressure is what would be expected to exceed autoregulatory reserve and produce watershed hypoperfusion (10, 11).

The lowest intraoperative MAP, by contrast, did not differ at all between patients with and without cognitive decline. If confirmed, this is clinically relevant: the single nadir value that is most salient during anaesthesia may be the least informative marker of cerebral risk, whereas the cumulative time below the threshold—which is not routinely displayed—appears more relevant.

### Age, collinearity, and the limits of what these data can support

Patients with cognitive decline were on average 30 years older than those without, and outcome events were confined to the oldest stratum. Age is an established determinant of perioperative neurocognitive vulnerability, acting through reduced cognitive reserve, cerebral small-vessel disease, and a rightward shift in the lower limit of autoregulation (10, 11, 22). Older patients are also more likely to have chronic hypertension and reduced vascular compliance and therefore both to be more haemodynamically labile under anaesthesia and to be more vulnerable to any given fall in perfusion pressure. In this cohort, a higher baseline MAP was itself associated with the outcome. This association admits two readings: it may mark the chronically hypertensive, autoregulation-shifted patient described above, or it may reflect the fact that baseline MAP is built into the severity and duration variables (Figure 5). These data cannot separate them, and the implausible magnitude of the unadjusted estimate (OR 1.99 per mmHg) is better read as a symptom of that entanglement than as an effect size.

The consequence is that our data cannot distinguish between three explanations: that prolonged hypotension causes early cognitive decline; that age causes both greater haemodynamic lability and greater cognitive vulnerability; or that both operate, with older patients being harmed by levels of hypotension that younger patients tolerate. The third is the most biologically plausible and is consistent with the wider literature, but our study cannot adjudicate between them. We deliberately refrain from presenting an adjusted effect estimate, because with 8 events, any such estimate would be an artefact of the modelling procedure rather than a finding.

### Interpreting the cognitive changes

Both instruments declined significantly at both postoperative time points, with large rank-based effect sizes. These statistics should not be over-read. The effect sizes are large because the direction of change was almost perfectly consistent—not one patient scored higher after surgery than before while the magnitude of change was under 0.7 points on a 30-point scale. With preoperative means of 29.5 and 29.0, both instruments were operating close to their ceiling, which mechanically prevents improvement and inflates the apparent consistency of decline. Statistical significance here reflects the sensitivity of a paired rank test in a large sample and not clinically meaningful cognitive injury.

Partial recovery by day 1 further suggests that much of the immediate change reflects residual anaesthetic effect, pain, opioid administration, fatigue, and disrupted sleep rather than neuronal injury. This is precisely why the 2018 consensus nomenclature reserves the diagnosis of postoperative neurocognitive disorder for impairment persisting beyond 30 days (1, 2), and why we have reported early decline rather than POCD throughout.

### Comparison with previous work and the Gulf context

Our observed incidence rate of 3.1% is at the low end of published estimates, which commonly range from 10% to 40% in elderly cohorts (3, 22). Three features of our cohort explain this: a mean age of 42 years with only 14% aged 60 or above; exclusion of patients with pre-existing cognitive impairment; and predominantly short procedures of low physiological severity. Our findings therefore describe a young, comparatively healthy surgical population and should not be extrapolated to elderly or high-risk cohorts. To our knowledge, this is among the first prospective studies of intraoperative hypotension and perioperative neurocognitive outcomes from the United Arab Emirates, and the young demographic profile of the surgical population in this region is itself a relevant observation for perioperative service planning.

### Clinical implications

Three implications follow, stated at the strength the data support. First, our findings suggest that the accumulated duration of hypotension may be more informative than whether a threshold was ever crossed, because the latter was uninformative here. However, because this association could not be separated from age, it requires confirmation in older cohorts with adequate adjustment before it can be used to guide intraoperative management. Second, individualised blood pressure targets may be particularly relevant in older patients and in those with elevated pre-induction MAP, in whom a given proportional fall represents a larger absolute departure from the accustomed perfusion pressure. Third, early postoperative cognitive screening, although feasible, requires instruments and time points capable of separating residual anaesthetic effect from genuine cognitive change; the MMSE and MoCA administered in recovery are not well-suited to this.

### Strengths and limitations

The principal strengths of the study are the prospective design with prespecified exposure and outcome definitions, complete three-time point cognitive assessment in all 261 analysed patients, the use of two complementary screening instruments, and the characterisation of hypotensive exposure by severity, duration, and pattern rather than occurrence alone.

### Limitations

This study has several limitations. The small number of participants with early postoperative cognitive decline (*n* = 8) limited statistical power and precluded meaningful multivariable adjustment; therefore, the regression findings should be considered only exploratory. Cognitive outcomes were assessed only during the immediate postoperative period and on postoperative day 1, representing early postoperative cognitive change rather than longer-term postoperative cognitive dysfunction. The MMSE and MoCA are screening instruments, and the use of the same test versions at each assessment means that practice effects cannot be excluded. In addition, although intraoperative anaesthetic and haemodynamic management data were available in the anaesthesia records, specific anaesthetic agents, vasopressors, intravenous fluids, and depth-of-anaesthesia parameters were not extracted or analysed because they were outside the predefined scope of the study. Blood pressure was recorded non-invasively at 5-min intervals rather than continuously; short episodes occurring between readings may have been missed, and episode durations had to be estimated from intermittent readings, and therefore, both cumulative duration and the ≤10 vs. >10 min pattern classification are subject to measurement error. Surgical risk within each specialty was not graded with a validated framework such as the Modified Johns Hopkins Surgical Criteria (23), and the surgical approach (open versus minimally invasive) and estimated blood loss were not recorded as study variables; each may influence intraoperative haemodynamics and early postoperative cognition and could therefore confound the associations reported. The threshold used to define early postoperative cognitive decline (>2 points) is pragmatic, and with near-ceiling baseline scores, a different cut-off could change the number of patients classified as having the outcome. Finally, the cognitive assessments were performed by the same researcher who recorded intraoperative blood pressure and was therefore not blinded to haemodynamic data; although standardised instruments were used, the possibility of assessment bias cannot be excluded. The single-centre design and exclusion of patients unable to complete postoperative day 1 assessment may also limit generalisability.

### Future research

Adequately powered studies in this field should recruit older and higher-risk cohorts in which the outcome is common enough to permit multivariable adjustment, extract haemodynamic data automatically to allow dose-based exposure metrics, assess cognition at 30 days and beyond using instruments with alternate forms and a reliable change index derived from a non-surgical control group, and report results under both relative and absolute definitions of hypotension so that the effect of the definition itself can be quantified.

## Conclusions

In a predominantly young cohort undergoing non-cardiac surgery, intraoperative hypotension defined as a>20% relative fall in MAP was near-universal and carried no discriminative value for early postoperative cognitive decline, whereas the severity, cumulative duration, and temporal pattern of hypotension were associated with the outcome. Early cognitive change after surgery was statistically significant but small and partially reversible by the first postoperative day. Because outcome events were confined to the oldest patients and numbered only eight, the association with hypotensive exposure cannot be separated from age and no causal claim is made. The most robust message is methodological: dichotomous relative definitions of intraoperative hypotension are poorly suited to neurocognitive research, and exposure should be quantified as a dose.

## Data Availability

The raw data supporting the conclusions of this article will be made available by the authors, without undue reservation.
